# Predictors of post-extubation stridor in patients on mechanical ventilation: a prospective observational study

**DOI:** 10.1038/s41598-021-99501-8

**Published:** 2021-10-07

**Authors:** Aiko Tanaka, Akinori Uchiyama, Yu Horiguchi, Ryota Higeno, Ryota Sakaguchi, Yukiko Koyama, Hironori Ebishima, Takeshi Yoshida, Atsuhiro Matsumoto, Kanaki Sakai, Daisuke Hiramatsu, Naoya Iguchi, Noriyuki Ohta, Yuji Fujino

**Affiliations:** 1 Department of Anesthesiology and Intensive Care, Osaka University Graduate School of Medicine, 2-15 Yamadaoka, Suita, Osaka 565-0871 Japan; 2 Division of Pediatrics, Osaka General Medical Center, 3-1-56 Bandai-Higashi, Sumiyoshi-ku, Osaka, 558-8558 Japan; 3 Division of Anesthesiology, Osaka General Medical Center, 3-1-56 Bandai-Higashi, Sumiyoshi-ku, Osaka, 558-8558 Japan; 4 Department of Anesthesiology, Kindai University Faculty of Medicine, 377-2, Ohno-Higashi, Osakasayama, Osaka 589-8511 Japan

**Keywords:** Respiration, Criticality, Respiratory tract diseases, Risk factors

## Abstract

The cuff leak test (CLT) has been widely accepted as a simple and noninvasive method for predicting post-extubation stridor (PES). However, its accuracy and clinical impact remain uncertain. We aimed to evaluate the reliability of CLT and to assess the impact of pre-extubation variables on the incidence of PES. A prospective observational study was performed on adult critically ill patients who required mechanical ventilation for more than 24 h. Patients were extubated after the successful spontaneous breathing trial, and CLT was conducted before extubation. Of the 191 patients studied, 26 (13.6%) were deemed positive through CLT. PES developed in 19 patients (9.9%) and resulted in a higher reintubation rate (8.1% vs. 52.6%, p < 0.001) and longer intensive care unit stay (8 [4.5–14] vs. 12 [8–30.5] days, p = 0.01) than patients without PES. The incidence of PES and post-extubation outcomes were similar in patients with both positive and negative CLT results. Compared with patients without PES, patients with PES had longer durations of endotracheal intubation and required endotracheal suctioning more frequently during the 24-h period prior to extubation. After adjusting for confounding factors, frequent endotracheal suctioning more than 15 times per day was associated with an adjusted odds ratio of 2.97 (95% confidence interval, 1.01–8.77) for PES. In conclusion, frequent endotracheal suctioning before extubation was a significant PES predictor in critically ill patients. Further investigations of its impact on the incidence of PES and patient outcomes are warranted.

## Introduction

Endotracheal intubation is an essential procedure for respiratory support in critically ill patients. However, complications resulting from mechanical trauma to the larynx by the endotracheal tube may lead to laryngeal edema^1,2^. Airflow through a narrowed upper airway manifests clinically as a post-extubation stridor (PES)^3^. The incidence of PES is 4–10% in patients who are endotracheally intubated for more than 24 h^4,5^. Patients with PES are likely to undergo increased reintubation^6,7^, which is associated with increased morbidity and mortality^8–10^.

To identify patients at risk of laryngeal edema, airway patency assessment before extubation has been evaluated. Since Miller and Cole evaluated 100 mechanically ventilated patients in 1996, the cuff leak test (CLT) has been widely used because of its noninvasiveness and avoidance of sophisticated equipment^11–15^. The cuff leak volume was defined as the tidal volume difference from before to after endotracheal tube (ETT) cuff deflation. The primary threshold of cuff leak volume of less than 110 mL was reported as the valid cut-off value for predicting PES with a positive predictive value of 0.80. Subsequent studies reported various cut-off values^16–18^. The other remarkable threshold, a cuff leak less than 10% of the tidal volume, was identified to predict PES or reintubation with a specificity of 96%^19^. In the previous meta-analyses of pooled studies on multiple CLT methods, the CLT displayed an accurate prediction for patients at high risk of the development of airway obstruction^20–22^ and is now widely used. However, some previous studies did not conduct a spontaneous breathing trial (SBT), which is the current standard weaning method, and there is concern that this may affect extubation outcomes. Indeed, recent studies have reported the poor diagnostic performance of the CLT in the current critical care settings^7,23,24^, and the diagnostic performance of CLT remains controversial.

Moreover, several studies related to baseline characteristics and airway management were performed to identify PES incidence risk factors. Important risk factors include female sex, body mass index (BMI), larger ETT diameter, and longer intubation duration^3,12,25–27^. However, each risk factor is insufficient to identify patients at high risk for PES when considered alone^3^, and the investigation of physiological or descriptive data at extubation is limited.

Accordingly, we aimed to determine the CLT value and the impact of pre-extubation variables for predicting PES among critically ill patients, based on the current standard and unified weaning readiness techniques.

## Methods

### Conduct of the study and selection criteria

This prospective study was performed in a multidisciplinary intensive care unit (ICU) of a tertiary care hospital between May 2017 and April 2019. The consecutive patients who had undergone invasive mechanical ventilation for more than 24 h and had been extubated following a successful SBT and assessment of the CLT were enrolled. Patients younger than 18 years of age, with a tracheostomy or unplanned extubation, who received palliative care or passed away under mechanical ventilation, and who passed away within 48 h after extubation were excluded. If patients received mechanical ventilation multiple times, the first extubation attempt after mechanical ventilation for more than 24 h was included.

This study was approved by the ethics review board of Osaka University Hospital (Approval Number: 19316), and the need for consent for research participation was waived. The study was performed in accordance with relevant guidelines and regulations.

### Assessment before extubation

According to the weaning strategies with national consensus, patients were considered eligible for SBT when the underlying cause of respiratory failure was resolved. Sufficient oxygenation (SpO_2_ > 90% at FiO_2_ ≤ 0.5 and positive end-expiratory pressure [PEEP] ≤ 8 cmH_2_O) and inspiratory effort (tidal volume > 5 mL/kg; minute volume < 15 L/min; rapid shallow breathing index < 105 breaths/min/L; and pH > 7.25) were verified prior to SBT. Clinicians suspended SBT if a patient had any of the following: significant deterioration of oxygenation compared to before SBT, respiratory rate ≥ 30 breaths/min, heart rate ≥ 140 beats/min, arrhythmia, myocardial ischemia symptom, sustained increased blood pressure, or appearance of respiratory distress as defined by paradoxical breathing, use of accessory muscles, sweating, or agitation. Patients were extubated if they tolerated 30 min of spontaneous breathing on PEEP of 5 cmH_2_O with pressure support (PS) of 5 cmH_2_O.

The cuff pressure was continuously measured and maintained at 25 cmH_2_O during mechanical ventilation in all patients. Prior to endotracheal extubation, CLT was performed by the attending intensivists. The patient was suctioned intraorally and intratracheally and placed in the assist-control ventilation mode. With the ETT cuff inflated to the occlusion volume, the average mechanical exhaled volume over the three consecutive respiratory cycles was measured and recorded. Subsequently, the ETT cuff was deflated. The mechanical exhaled tidal volume was monitored over the next six consecutive respiratory cycles to ensure the exclusion of erroneous values. The average of the three lowest exhaled tidal volumes was recorded. The lost tidal volume on exhalation, the cuff leak volume, was calculated as the tidal volume difference between the inflated and deflated ETT cuffs. Moreover, the cuff leak volume was divided by the tidal volume before cuff deflation and multiplied by 100^19^; the resulting value was regarded as the percent cuff leak. The high risk of upper airway obstruction was determined as the positive cuff leak result, the cuff leak volume ≤ 110 mL, and/or the percent cuff leak ≤ 10%, before extubation^11,19,21^.

### Data collection

Variables collected of each patient included age, sex, BMI, comorbidities, Acute Physiology and Chronic Health Evaluation (APACHE) II and III scores as indicators of disease severity on ICU admission, the reason for intubation, the size of the endotracheal tube, and the duration of mechanical ventilation. In addition, arterial blood gas values and ventilation data during successful SBT were recorded. Simultaneously, we collected information on patient management during the 24 h prior to extubation. Fluid balance, increased body weight when compared with the body weight prior to ICU admission, and as an indicator of tracheal–bronchial secretion, endotracheal suctioning, were recorded 24 h prior to extubation. Systemic steroid therapy was recorded if the patient was administered steroid ≥ 20 mg/day of methylprednisolone at least 4 h prior to extubation. Furthermore, the severity of organ dysfunction during extubation was assessed using the Sequential Organ Failure Assessment (SOFA) score^28^.

Moreover, the following data were recorded after extubation: reintubation and the incidence of PES within 48 h after extubation, ICU and hospital lengths of stay, and ICU and hospital mortality. Patients’ respiratory function and hemodynamics were closely monitored for 48 h after extubation. They were reintubated if they presented cardiac arrest, refractory hypoxemia, severe hemodynamic instability without response to fluids and vasoactive drugs, persistent inability to remove excessive secretions, upper airway obstruction, agitation, and loss of consciousness at the discretion of the intensivist in charge. A patient having PES was identified with the presence of respiratory distress with high-pitched inspiratory wheezing. The incidence of PES was assessed and recorded by clinicians and decisions concerning the medical treatment for PES were made by the attending intensivists.

### Statistical analyses

Categorical data are presented as numbers and percentages. Distributed data are presented as medians and interquartile ranges. Differences in proportions were evaluated using the chi-square test or Fisher’s exact test, and differences in distributed data with the Mann–Whitney U test for the two groups. A multiple logistic regression analysis model was used to determine PES incidence risk factors. Covariates with p < 0.05 in the univariate analysis were included in the multivariate model. To adjust for potential confounding factors, sex, BMI (> 25 kg/m^2^), and positive CLT were added to the model. All statistical inferences were made with a 2-sided significance level of 5% and conducted with R version 4.0.1 (2020, R Foundation for Statistical Computing, Vienna, Austria).

### Ethics declarations

This study was approved by the ethics review board of Osaka University Hospital (Approval Number: 19316), and the need for consent for research participation was waived.

## Results

**Figure 1:**
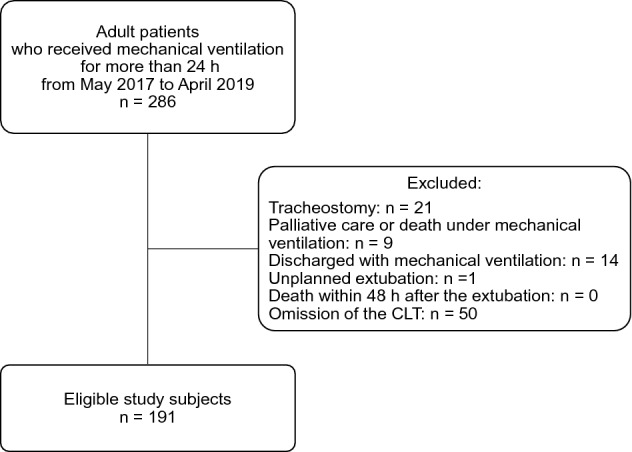
Flowchart of the study patients. *CLT* cuff leak test.

Over the 2-year study period, a total of 286 adult patients required invasive mechanical ventilation for more than 24 h. CLTs were not performed for 95 patients for the following reasons: tracheostomy (n = 21), palliative care or death under mechanical ventilation (n = 9), ICU discharge with mechanical ventilation (n = 14), unplanned extubation (n = 1), or omission of the CLT (n = 50). The remaining 191 adult patients were included in this study (Fig. 1). The characteristics of the study population are presented in Table 1. The median age was 65 years, and 67 (35.1%) patients were women. The main causes of ICU admission were surgical (75.9%). Reasons for mechanical ventilation included postoperative acute respiratory failure defined as ineligible decision for weaning and extubation in patients after surgery (n = 143; 74.9%), pneumonia (n = 16; 8.4%), cardiac failure (n = 10; 5.2%), and cardiac arrest (n = 8; 4.2%).

**Table 1:** Baseline characteristics according to the CLT result.

	All (n = 191)	Negative CLT (n = 165)	Positive CLT (n = 26)	p value
Age, years	65 (53–74)	65 (53–74)	65 (50–74)	0.81
Sex (female)	67 (35.1%)	49 (29.7%)	18 (69.2%)	< 0.001
BMI, kg/m^2^	22.5 (20.0–25.5) n = 189	22.2 (19.9–25.2) n = 163	25.3 (22.5–26.9) n = 26	0.01
BMI > 25 kg/m^2^	61 (32.3%) n = 189	46 (28.2%) n = 163	15 (57.7%) n = 26	0.01
APACHE II score	15 (12–19)	15 (12–19)	15 (12–20)	0.55
APACHE III score	60 (48–71)	60 (48–71)	58.5 (51–73)	0.70
Comorbidities				
Heart failure	72 (37.7%)	66 (40%)	6 (23.1%)	0.13
Asthma	7 (3.7%)	5 (3.0%)	2 (7.7%)	0.25
COPD	8 (4.2%)	8 (4.8%)	0 (0%)	0.60
Interstitial lung disease	12 (6.3%)	10 (6.1%)	2 (7.7%)	0.67
Surgical diagnosis	145 (75.9%)	123 (74.5%)	22 (84.6%)	0.33
Systematic diagnosis				
Cardiovascular	127 (66.5%)	111 (67.3%)	16 (61.5%)	0.48
Respiratory	25 (13.1%)	22 (13.3%)	3 (11.5%)	
Gastrointestinal	30 (15.7%)	25 (15.2%)	5 (19.2%)	
Neurology	1 (0.5%)	1 (0.6%)	0 (0%)	
Sepsis	2 (1.0%)	1 (0.6%)	1 (3.8%)	
Hematology	1 (0.5%)	1 (0.6%)	0 (0%)	
Renal/genitourinary	2 (1.0%)	2 (1.2%)	0 (0%)	
Gynecology	1 (0.5%)	1 (0.6%)	0 (0%)	
Other	2 (1.0%)	1 (0.6%)	1 (3.8%)	
Cause of mechanical ventilation				
Postoperative acute respiratory failure	143 (74.9%)	123 (74.5%)	20 (76.9%)	0.39
Pneumonia	16 (8.4%)	15 (9.1%)	1 (3.8%)	
Sepsis	5 (2.6%)	3 (1.8%)	2 (7.7%)	
Cardiac failure	10 (5.2%)	9 (5. 5%)	1 (3.8%)	
Coma	1 (0.5%)	1 (0.6%)	0 (0%)	
Cardiac arrest	8 (4.2%)	6 (3.6%)	2 (7.7%)	
Other	8 (4.2%)	8 (4.8%)	0 (0%)	
ETT diameter, mm (among male)	8 (8–8) n = 124	8 (8–8) n = 116	8 (7.9–8) n = 8	0.51
ETT diameter, mm (among female)	7 (7–7) n = 67	7 (7–7) n = 49	7 (7–7) n = 18	0.32

Data are presented as median and interquartile range or number (percentage).

The positive CLT was defined as the cuff leak volume ≤ 110 mL and/or the percent cuff leak ≤ 10%

*CLT* cuff leak test, *BMI* body mass index, *APACHE* acute physiology, and chronic health evaluation, *COPD* chronic obstructive pulmonary disease, *ETT* endotracheal tube.

### CLT result and its clinical impact

Twenty-six (13.6%) patients were determined to be positive through the CLT before extubation (Table 1). Positive CLT results were detected more often in female patients (18/67; 26.9%) than in male patients (8/124; 6.5%) (p < 0.001). Besides, patients with positive CLT results had a significantly larger BMI than their counterparts. The ETT diameter, duration of mechanical ventilation, and data from 24 h before extubation were similar regardless of CLT results (Tables 1, 2). The cuff leak volume and percent cuff leak were significantly smaller in patients with positive CLT results. All patients with positive CLT results had a cuff leak volume ≤ 110 mL; however, only 9 (34.6%) patients had a percent cuff leak ≤ 10%. The PES incidence was similar in the results of both CLT (negative: 9.7% vs. positive: 11.5%, p = 0.73) (Supplementary Table S1 shows this in more detail). Moreover, there were no differences in the reintubation rate, ICU and hospital lengths of stay, and ICU and hospital mortality according to the CLT results.

**Table 2:** Data at extubation and the detail of CLT.

	All	Negative CLT	Positive CLT	p value
SOFA score before extubation	8 (6–10)	8 (6–10)	8 (7–9)	0.77
Duration of mechanical ventilation, h	93 (45–197)	93 (46–194)	90 (40–231)	0.49
Arterial blood gas levels and respiratory data during the successful SBT				
pH	7.43 (7.40–7.46)	7.43 (7.40–7.46)	7.43 (7.40–7.46)	0.90
PaCO_2_, mmHg	41.4 (38.4–44.1)	41.3 (38.4–43.9)	41.8 (39.3–45.2)	0.48
PaO_2_/FiO_2_, mmHg	314 (253–379)	314 (257–387)	308 (247–359)	0.61
Rapid shallow breathing index, breaths/min/L	36 (27–50)	38 (28–50)	37 (27–48)	0.98
SpO_2_, %	99 (97–100)	99 (97–100)	98 (97–99)	0.22
Data during 24 h before extubation				
Increased body weight from ICU admission, kg	0.4 (− 1.1 to 2.1) n = 187	0.4 (− 1.2 to 2) n = 161	0.7 (− 0.3 to 2.7) n = 26	0.28
Number of endotracheal suctioning	14 (11–17)	14 (11–17.3)	13.5 (11.3–16.5)	0.56
Fluid balance, mL	− 365 (− 1222 to − 179)	− 365 (− 1204 to − 214)	− 94 (− 1151 to − 44)	0.71
Systemic steroid therapy	16 (8.4%)	12 (7.3%)	4 (15.4%)	0.24
Cuff leak test				
Cuff leak volume, mL	283 (184–391)	301 (224–412)	53 (12–93)	< 0.001
Percent cuff leak, %	57.3 (40.9–75.3)	61.9 (46.2–76.3)	11.5 (2.6–21.1)	< 0.001
Cuff leak volume ≤ 110 mL	26 (13.6%)	–	26 (100%)	–
Percent cuff leak ≤ 10%	9 (4.7%)	–	9 (34.6%)	–

Data are presented as median and interquartile range or number (percentage).

The positive CLT was defined as the cuff leak volume ≤ 110 mL and/or the percent cuff leak ≤ 10%

*CLT* cuff leak test, *SOFA* sequential organ failure assessment, *SBT* spontaneous breathing trial.

### Comparison of patients with and without PES

The overall incidence of PES was 9.9% (19/191). Table 3 compares the baseline characteristics of patients with and without PES. There were no significant differences in sex or BMI. The SOFA score at extubation, respiratory data, and the administration of systemic steroids during the 24-h period before extubation were similar in both groups (Table 4). Patients with PES had a longer duration of mechanical ventilation and required endotracheal suctioning more frequently than patients without PES. However, the CLT results were similar in both groups. As post-extubation outcomes, 8 (42.1%) patients with PES required reintubation for PES (Table 5). The reintubation rate for all causes was significantly higher in patients with PES than in those without PES (8.1% vs. 52.6%, p < 0.001). Moreover, the ICU length of stay was significantly longer for patients with PES than for those without PES (p = 0.01). There were no significant differences in hospital length of stay and ICU or hospital mortality.

**Table 3:** Characteristics of patients with or without PES.

	Patients without PES (n = 172)	Patients with PES (n = 19)	p value
Age, years	64 (52–74)	69 (62.5–74.5)	0.34
Sex (female)	60 (34.9%)	7 (36.8%)	1.00
BMI, kg/m^2^	22.5 (19.9–25.4) n = 170	23.7 (21.1–27.0) n = 19	0.28
BMI > 25 kg/m^2^	54 (31.4%) n = 170	7 (36.8%) n = 19	0.80
APACHE II score	15 (12–19)	14 (11.5–20)	0.83
APACHE III score	59 (48–71)	66 (49–74)	0.42
Comorbidities			
Heart failure	65 (37.8%)	7 (36.8%)	1.00
Asthma	5 (2.9%)	2 (10.5%)	0.15
COPD	7 (4.1%)	1 (5.3%)	0.58
Interstitial lung disease	10 (5.8%)	2 (10.5%)	0.35
Surgical diagnosis	133 (77.3%)	12 (63.2%)	0.17
Cause of mechanical ventilation			
Postoperative acute respiratory failure	131 (76.2%)	12 (63.2%)	0.186
Pneumonia	13 (7.6%)	3 (15.8%)	
Sepsis	3 (1.7%)	2 (10.5%)	
Cardiac failure	9 (5.2%)	1 (5.3%)	
Coma	1 (0.6%)	0 (0%)	
Cardiac arrest	7 (4.1%)	1 (5.3%)	
Other	8 (4.7%)	0 (0%)	
ETT diameter, mm (among male)	8 (8–8) n = 112	8 (8–8) n = 12	0.93
ETT diameter, mm (among female)	7 (7–7) n = 60	7 (7–7.3) n = 7	0.33

Data are presented as median and interquartile range or number (percentage).

*PES* post-extubation stridor, *BMI* body mass index, *APACHE* acute physiology, and chronic health evaluation, *COPD* chronic obstructive pulmonary disease, *ETT* endotracheal tube.

**Table 4:** Data at extubation among the patients with or without PES.

	Patients without PES	Patients with PES	p value
SOFA score at extubation	8 (6–10)	8 (7–10)	0.67
Duration of total mechanical ventilation, hour	90 (44–170)	194 (93–677)	0.01
Prolonged total mechanical ventilation > 4 days	80 (46.5%)	14 (73.7%)	0.03
Arterial blood gas levels and respiratory data during the successful SBT			
pH	7.43 (7.40–7.46)	7.43 (7.41–7.45)	0.64
PaCO_2_, mmHg	41.2 (38.4–43.9)	43.2 (40.9–46.2)	0.07
PaO_2_/FiO_2_, mmHg	313 (251–386)	314 (289–340)	0.61
Rapid shallow breathing index, breaths/min/L	38 (27–50)	39 (29–46)	0.97
SpO_2_, %	99 (97–100)	99 (98–99)	0.75
Data during 24 h before extubation			
Increased body weight from ICU admission, kg	0.4 (− 1 to 2.1) n = 169	0.4 (− 1.2 to 2.0) n = 18	0.86
Number of endotracheal suctioning	14 (11–17)	17 (13.5–20)	0.04
Endotracheal suctioning > 15 times	63 (36.6%)	13 (68.4%)	0.01
Fluid balance, mL	− 352 (− 1129 to 90)	− 526 (− 1305 to − 203)	0.30
Systemic steroid therapy	14 (8.1%)	2 (10.5%)	0.67
Cuff leak test			
Cuff leak volume ≤ 110 mL	23 (13.4%)	3 (15.8%)	0.73
Percent cuff leak ≤ 10%	7 (4.1%)	2 (10.5%)	0.22
Positive CLT	23 (13.4%)	3 (15.8%)	0.73

Data are presented as median and interquartile range or number (percentage).

The positive CLT was defined as the cuff leak volume ≤ 110 mL and/or the percent cuff leak ≤ 10%

*PES* post-extubation stridor, *SOFA* sequential organ failure assessment, *SBT* spontaneous breathing trial, *CLT* cuff leak test.

**Table 5:** Post-extubation outcomes of patients with and without PES.

	All	Patients without PES	Patients with PES	p value
Reintubation within 48 h, all cause	24 (12.6%)	14 (8.1%)	10 (52.6%)	< 0.001
Treatment of PES				
Intravenous steroid administration for post-extubation stridor	5/191 (26.2%)	–	5/19 (26.3%)	–
Adrenaline inhalation for post-extubation stridor	0 (0%)	–	0 (0%)	–
Reintubation for post-extubation stridor	8/191 (4.2%)	–	8/19 (42.1%)	–
ICU length of stay, day	8 (5–14)	8 (4.5–14)	12 (8–30.5)	0.01
Hospital length of stay, day	46 (28–80)	46 (28–80)	46 (28–81)	0.86
ICU mortality	6 (3.1%)	5 (2.9%)	1 (5.3%)	0.47
Hospital mortality	15 (7.9%)	12 (7.0%)	3 (15.8%)	0.18

Data are presented as median and interquartile range or number (percentage).

*PES* post-extubation stridor, *ICU* intensive care unit.

### Factors associated with PES

In the univariate logistic regression analysis, the positive result of CLT was not significantly associated with the incidence of PES, while there were differences in frequent endotracheal suctioning and prolonged mechanical ventilation (Table 6). After adjusting for confounding factors in the multivariate analysis, the frequent endotracheal suctioning more than 15 times during the 24-h period before extubation remained significant (adjusted odds ratio 2.97; 95% confidence interval [CI] 1.01–8.77).

**Table 6:** Univariate and multivariate logistic regression analyses for PES.

	Univariate analysis		Multivariate analysis	
	Odds ratio (95% CI)	p value	Adjusted odds ratio* (95% CI)	p value
Positive CLT	1.21 (0.33–4.5)	0.77		
Endotracheal suctioning > 15 times during the 24-h period prior to the extubation	3.71 (1.34–10.3)	0.01	2.97 (1.01–8.77)	0.048
Prolonged mechanical ventilation > 4 days	3.22 (1.11–9.33)	0.03	2.43 (0.79–7.42)	0.12

*PES* post-extubation stridor, *CI* confidence interval, *CLT* cuff leak test.

*Adjusted by gender, BMI > 25 kg/m^2^, and positive CLT.

## Discussion

We conducted a prospective cohort study to evaluate the performance of CLT and the potential impact of pre-extubation variables on the incidence of PES in critically ill patients who were extubated after successful SBT under the unified ventilatory support setting. Though the patients with positive CLT results had significantly smaller cuff leak volumes and percent cuff leaks than those with negative CLT results, the performance of the CLT was insufficient for the accurate prediction of PES. The potential risk factors, female sex, and the larger BMI were associated with CLT results. However, these factors were not significantly associated with the incidence of PES. Frequent endotracheal suctioning during the 24-h period prior to extubation was independently associated with the incidence of PES.

As the direct visualization of laryngeal edema is difficult with an endotracheal tube in position, the CLT has been widely accepted to predict PES incidence. However, this study contributes to the limited and conflicting evidence regarding the predictive accuracy of CLT. The standard rate of patients deemed positive through the CLT (the cuff leak volume ≤ 110 mL) has been reported to be between 5 and 30% in critically ill adult settings^11,20,23,27^, which validates our finding of 13.6%. Simultaneously, the incidence of PES in this study was 9.9%, which was comparable to the results of previous studies with a lower rate than the positive CLT rate. Thus, the relatively low prevalence of PES has been regarded as a possible factor for the poor performance of the CLT in the identification of patients at high risk of PES occurrence^3^. Laryngeal edema should occur in most intubated patients with varying degrees. The quantity of data on airway stenosis regarding PES and respiratory distress incidence is limited^29–31^. Furthermore, after prolonged endotracheal intubation, bronchoscopy showed that the tracheal granulation tissue or ulcerations spread widely from the vocal cord to the previous ETT balloon cuff site^17,32^. As global guidelines have not standardized the ETT size based on the tracheal diameter^33,34^, our patients were provided a similar ETT size regardless of the CLT results. Mild laryngeal edema encompassing the ETT balloon cuff could be demonstrated by the CLT without significant clinical symptoms, leading to an insufficient interpretation of the test result^24^. Moreover, the cumulative evidence for the predictive accuracy of CLTs was based on a variety of mechanical ventilation modes and clinical settings during CLT^22^. Following the reference report by Miller et al.^11^, the assist-control ventilation mode was commonly used, just as in this study^35^. However, the fact that rigorous and reproducible CLT assessment may require firm sedation and paralysis to avoid erroneous measurement caused by coughing or agitation^36^ and the potential influence of glossoptosis on the head position are challenges for the current standard CLT method^37^.

Furthermore, systemic steroid therapy before elective extubation has been associated with significant PES incidence reductions^5,25,38,39^. A recent meta-analysis including 11 trials of 2472 participants revealed that prophylactic systemic steroid therapy was associated with a reduced incidence of post-extubation airway events (risk ratio [RR], 0.43; 95% CI 0.29–0.66) and reintubation (RR, 0.42; 95% CI 0.25–0.71) when compared with placebo or no treatment^29,40^. The authors also screened six trials that documented the adverse effects and concluded that the short duration of systemic steroid administration rarely resulted in severe adverse effects. There was no hyperglycemia or gastrointestinal bleeding incidence, and only one patient who received systemic steroids was recognized with the infection. Thus, the current guidelines indicate the use of prophylactic systemic steroids in patients with airway compromise^12,41^. In our cohort, patients received systemic steroid therapy for at least 4 h before extubation for all causes (7.3% and 15.4% of the patients with negative and positive CLT results, respectively). Systemic steroid therapy was not individually associated with PES incidence (p = 0.67); however, it may potentially treat laryngeal edema and influence patient outcomes.

Multiple investigations of the risk factors for the incidence of PES have been reported, with conflicting conclusions. In this study, female sex and larger BMI were associated with CLT results. Patients with PES had a longer duration of intubation and frequent endotracheal suctioning than those without PES. However, multivariate analysis revealed that frequent endotracheal suctioning during the 24-h period before extubation was independently associated with PES incidence in our patient population. The number of endotracheal suctioning indicates the amount of trachea-bronchial secretion. Excessive tracheal-bronchial secretions can lead to bronchial plugging and atelectasis, which can result in respiratory failure. Recent reports substantiated the significance of excessive tracheal-bronchial secretions before extubation as risk factors for reintubation^8,42–44^. In addition, the tracheal-bronchial secretions are relevant indicators of laryngeal edema because they are triggered simultaneously, and both are caused by airway inflammation^45^. Furthermore, acute venous congestion due to excessive fluid infusion could increase pulmonary congestion causing tracheal-bronchial secretions^46,47^; moreover, it could also increase the risk of laryngeal edema^48^. Subsequently, the procedure of endotracheal suctioning itself has been reported to potentially cause tracheal-bronchial inflammation^49,50^ and pulmonary edema^51^. Thus, laryngeal edema and tracheal-bronchial secretions are caused in conjunction with each other, however, their relative importance has not been sufficiently investigated. The only previous study to research the association between the number of endotracheal suctioning and laryngeal injury was in 1981, in a single prospective cohort of 150 critically ill patients who received mechanical ventilation with endotracheal intubation or tracheostomy^2^. Stauffer et al. reported that endotracheal suctioning frequency was not significantly associated with total laryngeal injury at autopsy. However, this analysis included 51 patients (34%) with a tracheostomy, and the liberation process was not standardized. According to the current clinical practice and the unified weaning readiness techniques, the present data provide a reasonable impact.

Our findings imply that a positive result of CLT is relatively common in critically ill patients who have undergone invasive mechanical ventilation for more than 24 h. However, they also imply that the CLT results are not significantly associated with PES incidence, which results in an increased reintubation rate and ICU length of stay. Despite the lack of a significant impact on demographic variables, we found that frequent endotracheal suctioning was an independent risk factor for PES incidence. This finding provides a rationale for further investigations of the risk management of upper airway obstruction.

Our study has several strengths. Relatively few studies have assessed the physiological or descriptive data at extubation as risk factors for PES incidence. Though the population we investigated was relatively small, our prospective cohort was representative, as the rate of positive results of CLT and the incidence of PES were comparable to those described in other recent studies. In addition, the rigorous SBT and CLT assessment revealed a robust evaluation of CLT and risk factors for the prediction of PES.

Some limitations of this study should be acknowledged. First, it is a single-center study, with all the limitations inherent in such a design. The results may not be generalizable to all geographical regions. Second, we employed the SBT method with pressure support ventilation (PSV) using low PS and PEEP levels, following the international guideline that recommends inspiratory pressure augmentation^52^. General SBT methods, both PSV and T-piece, were reported to have comparable predictive performance for successful extubation^53^. However, low level of assistance without PEEP has recently been recommended as a SBT method^54^, which is distinct from the method used in this study. Third, based on previous studies reporting PES rates of 4–10%, we conducted a 2-year observational study to obtain at least 10 patients presenting with PES in an ICU with approximately 50–150 eligible patients per year to clarify its predictive performance using binary logistic regression analysis. To detect the association between the CLT results and PES incidence with 95% certainty at 80% power, we assumed that a total of 184 patients would be required. This was based on CLT positivity and PES incidence rates of 18% and 4%, respectively, according the results of a previous study that included the largest number of patients^27^. We found that that 19 among 191 study patients had PES and could accumulate sufficient cases for analysis, however, the CLT positivity rate and the incidence of PES in this study suggested that a greater number of patients were warranted to determine the predictive ability of CLT for PES. Hence, our results should be examined in a meta-analysis and in a pooled analysis. In addition, this study included all patients with mechanical ventilation and not exclusively patients at high risk for PES, for which the guideline recommends CLT^12^. Furthermore, patients with unplanned extubation regarded as high risk for PES^12^ were not included in the study due to lack of SBT or CLT, and we did not identify any patient who was reintubated after unplanned extubation and then underwent planned extubation. Forth, the formal PES diagnosis was based on clinical symptoms and, therefore, relatively subjective. Fifth, although the assessment of SBT and CLT was standardized, the final decision of extubation, the administration of systemic steroids, the treatment of patients with PES, and the decision of reintubation were at the discretion of the clinician in charge of the patients without specific institutional protocols.

## Conclusions

In the critically ill patients receiving mechanical ventilation, patients who required frequent endotracheal suctioning were at a high risk of developing PES. Though its impact on the incidence of PES and patient outcomes needs to be further explored, frequent endotracheal suctioning before extubation could be added to the usual prediction for PES.

## Supplementary Information

Supplementary Information.

## Data Availability

The datasets generated during and/or analyzed during the current study are available from the corresponding author on reasonable request.
