# Supplementary material for: Predictors of post-extubation stridor in patients on mechanical ventilation: a prospective observational study
Source: Sci Rep. 2021 Oct 7;11:19993. doi: 10.1038/s41598-021-99501-8 (PMC8497593; doi:10.1038/s41598-021-99501-8)
Supplement: Supplementary file 1 — Supplementary Information. [file 41598_2021_99501_MOESM1_ESM.pdf]

Supplementary Information

Predictors of Post-Extubation Stridor in Patients on Mechanical Ventilation: A Prospective Observational Study

Authors:

Aiko Tanaka [1*], Akinori Uchiyama [1], Yu Horiguchi [1], Ryota Higeno [2], Ryota Sakaguchi [1], Yukiko Koyama [1], Hironori Ebishima [1], Takeshi Yoshida [1], Atsuhiro Matsumoto [3], Kanaki Sakai [1], Daisuke Hiramatsu [1], Naoya Iguchi [1], Noriyuki Ohta [4], and Yuji Fujino [1]

Supplementary Table S1 Post-extubation outcomes according to the CLT result

	All (n = 191)	Negative CLT (n = 165)	Positive CLT (n = 26)	p value
Reintubation within 48 h, all cause	24 (12.7%)	22 (13.3%)	2 (7.7%)	0.63
Post-extubation stridor	19 (9.9%)	16 (9.7%)	3 (11.5%)	0.73
Intravenous steroid administration for post-extubation stridor	5 / 19 (26.3%)	5 / 16 (3.0%)	0 / 3 (0%)	0.53
Adrenaline inhalation for post-extubation stridor	0 (0%)	0 (0%)	0 (0%)	-
Reintubation for post-extubation stridor	8 / 19 (42.1%)	7 / 16 (43.8%)	1 / 3 (33.3%)	1.00
ICU length of stay, day	8 (5 - 14)	8 (5 - 14)	9 (4.3 - 16.3)	0.75
Hospital length of stay, day	46 (28 - 80)	49 (28 - 94)	40 (22 - 52)	0.12
ICU mortality	6 (3.1%)	5 (3.0%)	1 (3.8%)	0.59
Hospital mortality	15 (7.9%)	14 (8.5%)	1 (3.8%)	0.70

Data are presented as median and interquartile range or number (percentage).

The positive CLT was defined as the cuff leak volume ≤ 110mL and / or the percent cuff leak ≤ 10%.

CLT, cuff leak test; ICU, intensive care unit
